# Improving the Driving of Community-Dwelling Older Canadians: A Randomized Controlled Trial

**DOI:** 10.1093/geroni/igaa057.2594

**Published:** 2020-12-16

**Authors:** Brenda Vrkljan, Ruheena Sangrar, Lauren Griffith, Lori Letts, Michelle Porter

**Affiliations:** 1 McMaster University, Hamilton, Ontario, Canada; 2 University of Manitoba, Winnipeg, Manitoba, Canada

## Abstract

Older Canadians, similar to aging drivers in many other countries, want to drive, need to drive, and live in communities where driving is both valued and necessary for out-of-home participation. Many community-dwelling seniors are medically fit-to-drive, yet their collision risk remains higher than most other age groups, which some have attributed to their propensity to drive shorter distances in high-traffic areas (Antin et al., 2017). In this randomized controlled trial, the effect of a customized video-based older driver training program on behind-the-wheel performance was captured using the latest technology for an on-road evaluation. Results indicated the mean reduction in number of driving errors [mean (95% CI)=-12.0(-16.5, -7.6),p<0.001] favoured the intervention group where their change between baseline and 4-week follow-up was statistically significant [mean(95% CI)=-10.3(-13.8, -6.8),p<0.001], but not for the control group [mean (95% CI)=1.7(-0.08, 4.2), p>0.05]. Our novel, video-based approach that provided individualized feedback improved driving performance for older drivers. Part of a symposium sponsored by Transportation and Aging Interest Group.

